# Vinegar as a Protective against Small-Pox

**Published:** 1873-11

**Authors:** 


### VINEGAR AS A PROTECTIVE AGAINST SMALL-POX.

Dr. Max Heller (Med. and Surg. Reporter) writes: Adults and half-grown persons, who were for a considerable time in the room with a patient during the development of small-pox, consequently breathing the same air, and even assisting him or her, by the *use of vinegar* either escaped altogether or if they were attacked from the tenth to the thirteenth day with fever, lassitude, pain in the limbs, etc., in consequence of which they were confined to bed, these symptoms lasted only from two to three days, after which they felt well again with the exception of some fatigue, and from one to four small pustules the size of a pin-head appeared on the face or upon the arms. Moreover, no injurious effect upon the general health was experienced, not even in children, to whom it was given by the teaspoonful, in consequence of the over-anxiety, without orders.

Those who took the vinegar irregularly were attacked on the tenth or fourteenth day with fever, pain in the limbs, etc., more severely than those who took it as directed, but they recovered in four to seven days; afterwards about eighty pustules, scarcely one-fourth the size of the ordinary ones, made their appearance, and these dried completely up in from three to six days, leaving a bluish-red spot, which also disappeared rapidly. Even in these cases the infection is undoubtedly proven, and the use of vinegar did not *accomplish altogether* a satisfactory result; nevertheless there was a great gain for those persons in having passed through the infection in from four to seven days, to a healthy condition. The spread of the fatal malady was thus considerably modified.

No doubt many persons are protected by vaccination, re-vaccination, and partially by an indisposition to the disease, even if they do breathe the same air, or come in actual contact with a patient during the stage of efflorescence; it is therefore doubtful whether those who drank the vinegar and were exempt from the disease are to be considered liable to have been infected; but the infection appears indisputable in those who drank the vinegar and after ten or fourteen days became ill in the manner above described, and on the third day of the illness from one to six abortive pustules regularly appeared on the face or upon the hands. To those the use of vinegar was of incalculable gain, and in those whose ready receptivity or non-receptivity could not have been recognized, the use of the vinegar was harmless.

All persons who came in direct or indirect contact with a small-pox patient were subjected to the vinegar treatment, and with much benefit. Healthy adults were ordered two tablespoonfuls of common vinegar, either with or without water, to be taken one hour after breakfast and towards evening for fourteen days; for half-grown or particularly delicate persons three-fourths of a tablespoonful once or twice daily will suffice. They should avoid the sick room as much as possible, enjoy plenty of fresh air, and guard against cold; the sick chamber is to be fumigated with vinegar vapors twice daily.

He also recommends to persons who communicate much with the public, as people in large cities, to take now and then a tablespoonful of vinegar for a week.

